# Dichlorido{μ-6,6′-dimeth­oxy-2,2′-[*o*-phenyl­enebis(nitrilo­methyl­idyne)]diphenolato}(dimethyl sulfoxide)lead(II)zinc(II) *N*,*N*-dimethyl­formamide solvate

**DOI:** 10.1107/S1600536808034338

**Published:** 2008-10-25

**Authors:** Hailong Wang, Daopeng Zhang, Laijin Tian, Li-Fang Zhang

**Affiliations:** a School of Chemistry & Chemical Technology, Shandong University, Jinan 250100, People’s Republic of China

## Abstract

In the heterodinuclear complex of the title compound,  [PbZn(C_22_H_18_N_2_O_4_)Cl_2_(C_2_H_6_OS)]·C_3_H_7_NO, the Zn^II^ atom is coordinated in a distorted square-pyramidal geometry by two N atoms and two O atoms from the diphenolate ligand, and one Cl atom which occupies the apical position. The Pb^II^ atom is coordinated in a distorted octa­hedral geometry by the four O atoms of the diphenolate ligand, one O atom from the dimethyl sulfoxide mol­ecule and one Cl atom. The dimethyl sulfoxide mol­ecule is disordered over two positions, with site occupancies of 0.576 (2) and 0.424 (2).

## Related literature

For general background, see: Karlin (1993 ▶); Ni *et al.* (2005 ▶); Ward (2007 ▶). For a related structure, see: Korupoju *et al.* (2000 ▶). For related literature on the preparative method, see: Lo *et al.* (2004 ▶); Sui *et al.* (2007 ▶).
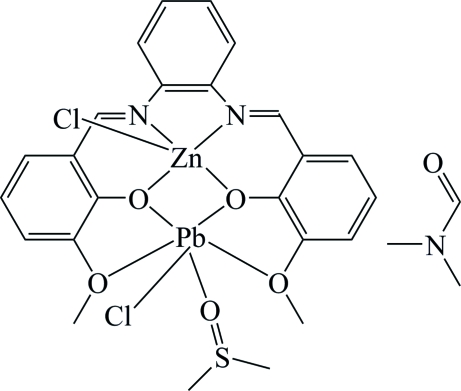


## Experimental

### 

#### Crystal data

[PbZn(C_22_H_18_N_2_O_4_)Cl_2_(C_2_H_6_OS)]·C_3_H_7_NO
*M* *_r_* = 869.07
Monoclinic, 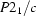

*a* = 15.2850 (7) Å
*b* = 18.8433 (8) Å
*c* = 10.7343 (5) Å
β = 94.771 (1)°
*V* = 3081.0 (2) Å^3^
*Z* = 4
Mo *K*α radiation
μ = 6.52 mm^−1^
*T* = 295 (2) K
0.2 × 0.15 × 0.1 mm

#### Data collection

Bruker APEX CCD area-detector diffractometer
Absorption correction: multi-scan (**SADABS**; Sheldrick, 2003 ▶) *T*_min_ = 0.343, *T*_max_ = 0.518
18055 measured reflections
6997 independent reflections
4923 reflections with *I* > 2σ(*I*)
*R*_int_ = 0.039

#### Refinement

*R*[*F* ^2^ > 2σ(*F* ^2^)] = 0.036
*wR*(*F* ^2^) = 0.073
*S* = 0.99
6997 reflections
387 parameters
28 restraints
H-atom parameters constrained
Δρ_max_ = 0.75 e Å^−3^
Δρ_min_ = −0.61 e Å^−3^

### 

Data collection: *SMART* (Bruker, 2001 ▶); cell refinement: *SAINT* (Bruker, 2001 ▶); data reduction: *SAINT*; program(s) used to solve structure: *SHELXS97* (Sheldrick, 2008 ▶); program(s) used to refine structure: *SHELXL97* (Sheldrick, 2008 ▶); molecular graphics: *SHELXTL* (Sheldrick, 2008 ▶); software used to prepare material for publication: *SHELXTL*.

## Supplementary Material

Crystal structure: contains datablocks global, I. DOI: 10.1107/S1600536808034338/is2345sup1.cif

Structure factors: contains datablocks I. DOI: 10.1107/S1600536808034338/is2345Isup2.hkl

Additional supplementary materials:  crystallographic information; 3D view; checkCIF report

## Figures and Tables

**Table 1:** Selected bond lengths (Å)

Zn1—N1	2.070 (4)
Zn1—N2	2.077 (3)
Zn1—O3	2.028 (3)
Zn1—O2	2.014 (3)
Zn1—Cl2	2.2686 (14)
Pb1—O1	2.791 (4)
Pb1—O2	2.476 (3)
Pb1—O3	2.420 (3)
Pb1—O4	2.667 (3)
Pb1—O5	2.741 (5)
Pb1—O5′	2.746 (7)
Pb1—Cl1	2.6088 (15)

**Fig. 1.**
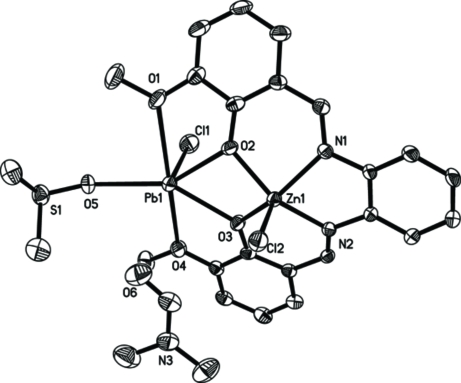
A view of (I) with the unique atom-labelling scheme. Displacement ellipsoids are drawn at the 30% probability level, all hydrogen atoms are omitted for clarity. Only one conpoment of the disordered DMSO molecule is shown.

## supplementary crystallographic information

### Comment

Heterometallic dinuclear complexes have been intensively studied owing to their unique physical and chemical properties (Ward, 2007; Ni *et al.*, 2005). In addition, these compounds exist at the active sites of many metalloenzymes and play important roles in biological systems (Karlin, 1993). Whereas, it is necessary to further widen the system of application of heterometallic compounds. Herein, a novel heterometallic binuclear Zn^II^Pb^II^ compound has been obtained by step-by-step method and its structure is depicted.

As shown in Fig. 1, the title compound is a binuclear neutral complex with a slightly distorted planar configuration. Each Zn^II^ atom is coordinated in a square-pyramidal geometry with the basal square formed by two N atoms and two O atoms from the *L* ligand, and with the apical position occupied by terminal Cl atom. The coordination environment of each Pb^II^ atom is in a distorted octahedral geometry composed of four O atoms from the ligand, one Cl atom and one O atom of the DMSO molecule. The Zn^II^ atom and the Pb^II^ atom are connected *via* two bridging O atoms of the ligand. The bond lengths of Zn—O, Zn—N and Zn—Cl are normal (Korupoju *et al.*, 2000).

### Experimental

The H_2_L ligand and the complex ZnL were synthesized according to the previous literatures (Lo *et al.*, 2004; Sui *et al.*, 2007). The title compound was obtained by allowing the mixture of ZnL (0.088 g, 0.2 mmol) and PbCl_2_.2H_2_O (0.063 g, 0.2 mmol) being refluxed in a DMF and DMSO (1:1) solution, cooled down to room temperature, then filtered. Yellow single crystals suitable for X-ray diffraction were obtained *via* slow evaporation of the filtrate at room temperature.

### Refinement

All H atoms bound to C were refined using a riding model, with distance C—H = 0.93 Å and *U*_iso_(H) = 1.2*U*_eq_(C) for aromatic atoms, and C—H = 0.96 Å and *U*_iso_(H) = 1.5*U*_eq_(C) for methyl atoms. The DMSO molecule was found to be disordered over two positions in a difference Fourier map. All the atoms were refined in two parts and the site occupancy factors were refined to 0.576 (2) (for atoms S1, O5, C26 and C27) and 0.424 (2) (for atoms S1', O5', C26' and C27'). The bonds S—O and S—C were restrained to 1.45 (1) and 1.82 (1) Å, respectively. The displacement parameters of each pair of disorder atoms were set to equal by the EADP instruction. The bond lengths C26—S1, C26'—S1', C27—S1 and C27'—C1' were restrained to be nearly equal by the SADI command with deviation 0.01 Å. The distances of S1—O5 and Zn1—O5 were also restrained nearly equal to that of S1'—O5' and Zn1—O5', respectively. The displacement parameter restraints (DELU) were applied to the disorder atoms (S1, C27, S1' and C27'). Additionally, atoms C27 and C27' were restrained to be approximately isotropic by the ISOR instruction.

### Crystal data

**Table:**

[PbZn(C_22_H_18_N_2_O_4_)Cl_2_(C_2_H_6_OS)]·C_3_H_7_NO	*F*(000) = 1696
*M**_r_* = 869.07	*D*_x_ = 1.874 Mg m^−^^3^
Monoclinic, *P*2_1_/*c*	Mo *K*α radiation, λ = 0.71073 Å
Hall symbol: -P2ybc	Cell parameters from 5739 reflections
*a* = 15.2850 (7) Å	θ = 1.7–27.5°
*b* = 18.8433 (8) Å	µ = 6.52 mm^−^^1^
*c* = 10.7343 (5) Å	*T* = 295 K
β = 94.771 (1)°	Block, yellow
*V* = 3081.0 (2) Å^3^	0.2 × 0.15 × 0.1 mm
*Z* = 4	

### Data collection

**Table:**

Bruker APEX CCD area-detector diffractometer	6997 independent reflections
Radiation source: fine-focus sealed tube	4923 reflections with *I* > 2σ(*I*)
graphite	*R*_int_ = 0.039
ω scans	θ_max_ = 27.5°, θ_min_ = 1.7°
Absorption correction: multi-scan (*SADABS*; Sheldrick, 2003)	*h* = −19→15
*T*_min_ = 0.343, *T*_max_ = 0.518	*k* = −23→24
18055 measured reflections	*l* = −13→12

### Refinement

**Table:**

Refinement on *F*^2^	Primary atom site location: structure-invariant direct methods
Least-squares matrix: full	Secondary atom site location: difference Fourier map
*R*[*F*^2^ > 2σ(*F*^2^)] = 0.036	Hydrogen site location: inferred from neighbouring sites
*wR*(*F*^2^) = 0.073	H-atom parameters constrained
*S* = 0.99	*w* = 1/[σ^2^(*F*_o_^2^) + (0.0296*P*)^2^] where *P* = (*F*_o_^2^ + 2*F*_c_^2^)/3
6997 reflections	(Δ/σ)_max_ = 0.001
387 parameters	Δρ_max_ = 0.75 e Å^−^^3^
28 restraints	Δρ_min_ = −0.61 e Å^−^^3^

### Special details

**Table:**

Geometry. All e.s.d.'s (except the e.s.d. in the dihedral angle between two l.s. planes) are estimated using the full covariance matrix. The cell e.s.d.'s are taken into account individually in the estimation of e.s.d.'s in distances, angles and torsion angles; correlations between e.s.d.'s in cell parameters are only used when they are defined by crystal symmetry. An approximate (isotropic) treatment of cell e.s.d.'s is used for estimating e.s.d.'s involving l.s. planes.
Refinement. Refinement of *F*^2^ against ALL reflections. The weighted *R*-factor *wR* and goodness of fit *S* are based on *F*^2^, conventional *R*-factors *R* are based on *F*, with *F* set to zero for negative *F*^2^. The threshold expression of *F*^2^ > σ(*F*^2^) is used only for calculating *R*-factors(gt) *etc*. and is not relevant to the choice of reflections for refinement. *R*-factors based on *F*^2^ are statistically about twice as large as those based on *F*, and *R*- factors based on ALL data will be even larger.

### Fractional atomic coordinates and isotropic or equivalent isotropic displacement parameters (Å^2^)

**Table:**

	*x*	*y*	*z*	*U*_iso_*/*U*_eq_	Occ. (<1)
C1	0.9922 (4)	0.5643 (3)	−0.2068 (8)	0.092 (2)	
H1A	1.0047	0.6013	−0.1464	0.138*	
H1B	0.9834	0.5848	−0.2888	0.138*	
H1C	1.0406	0.5318	−0.2041	0.138*	
C2	0.8849 (3)	0.4724 (3)	−0.2534 (5)	0.0464 (12)	
C3	0.9128 (4)	0.4565 (3)	−0.3684 (5)	0.0594 (15)	
H3	0.9561	0.4841	−0.4004	0.071*	
C4	0.8775 (4)	0.4007 (3)	−0.4364 (5)	0.0615 (16)	
H4	0.8966	0.3910	−0.5147	0.074*	
C5	0.8147 (3)	0.3591 (3)	−0.3907 (5)	0.0535 (14)	
H5	0.7913	0.3212	−0.4380	0.064*	
C6	0.7850 (3)	0.3730 (2)	−0.2721 (4)	0.0384 (11)	
C7	0.8183 (3)	0.4316 (2)	−0.2022 (4)	0.0396 (11)	
C8	0.7195 (3)	0.3250 (2)	−0.2308 (4)	0.0387 (11)	
H8	0.6966	0.2912	−0.2876	0.046*	
C9	0.6238 (3)	0.2772 (2)	−0.0917 (4)	0.0340 (10)	
C10	0.6124 (3)	0.2101 (2)	−0.1400 (4)	0.0458 (12)	
H10	0.6504	0.1929	−0.1964	0.055*	
C11	0.5449 (3)	0.1680 (3)	−0.1049 (5)	0.0503 (13)	
H11	0.5370	0.1227	−0.1386	0.060*	
C12	0.4890 (3)	0.1927 (3)	−0.0202 (5)	0.0497 (13)	
H12	0.4429	0.1643	0.0018	0.060*	
C13	0.5009 (3)	0.2587 (2)	0.0317 (4)	0.0440 (12)	
H13	0.4636	0.2747	0.0898	0.053*	
C14	0.5687 (3)	0.3017 (2)	−0.0023 (4)	0.0338 (10)	
C15	0.5294 (3)	0.4084 (2)	0.0968 (4)	0.0329 (10)	
H15	0.4743	0.3883	0.1023	0.040*	
C16	0.5433 (3)	0.4791 (2)	0.1456 (4)	0.0333 (10)	
C17	0.4754 (3)	0.5081 (3)	0.2123 (4)	0.0414 (12)	
H17	0.4262	0.4807	0.2241	0.050*	
C18	0.4806 (3)	0.5746 (3)	0.2589 (5)	0.0493 (13)	
H18	0.4371	0.5916	0.3067	0.059*	
C19	0.5518 (3)	0.6180 (2)	0.2349 (4)	0.0429 (12)	
H19	0.5542	0.6645	0.2638	0.051*	
C20	0.6174 (3)	0.5923 (2)	0.1696 (4)	0.0377 (11)	
C25	0.7952 (4)	0.5309 (4)	0.3414 (6)	0.0688 (17)	
H25	0.7753	0.4877	0.3083	0.083*	
C23	0.7891 (5)	0.6193 (4)	0.4986 (6)	0.105 (3)	
H23A	0.7556	0.6292	0.5683	0.158*	
H23B	0.7822	0.6575	0.4395	0.158*	
H23C	0.8500	0.6145	0.5274	0.158*	
C24	0.6895 (4)	0.5155 (4)	0.4933 (6)	0.092 (2)	
H24A	0.6710	0.5410	0.5639	0.138*	
H24B	0.7109	0.4696	0.5197	0.138*	
H24C	0.6406	0.5101	0.4318	0.138*	
C21	0.6168 (3)	0.5213 (2)	0.1249 (4)	0.0343 (10)	
C22	0.6974 (4)	0.7029 (3)	0.1830 (6)	0.0630 (16)	
H22A	0.7504	0.7230	0.1565	0.095*	
H22C	0.6992	0.7041	0.2726	0.095*	
H22B	0.6479	0.7298	0.1483	0.095*	
N1	0.6903 (2)	0.32535 (18)	−0.1215 (3)	0.0351 (9)	
N2	0.5875 (2)	0.37078 (18)	0.0461 (3)	0.0337 (8)	
N3	0.7585 (3)	0.5545 (3)	0.4398 (5)	0.0637 (13)	
O3	0.6844 (2)	0.49968 (15)	0.0660 (3)	0.0412 (8)	
O2	0.7936 (2)	0.45072 (16)	−0.0930 (3)	0.0452 (8)	
O1	0.9148 (2)	0.52743 (19)	−0.1786 (4)	0.0606 (10)	
O6	0.8536 (3)	0.5608 (3)	0.2891 (5)	0.0903 (15)	
O4	0.6892 (2)	0.63108 (17)	0.1411 (3)	0.0521 (9)	
Zn1	0.71596 (3)	0.39911 (3)	0.01970 (5)	0.03524 (13)	
Pb1	0.805850 (13)	0.569995 (10)	0.003745 (18)	0.04440 (7)	
Cl1	0.70822 (10)	0.63063 (8)	−0.17678 (14)	0.0672 (4)	
Cl2	0.80237 (8)	0.34936 (7)	0.17826 (12)	0.0528 (3)	
C27	0.9574 (13)	0.7236 (9)	0.2609 (7)	0.098 (3)	0.576 (2)
H27A	0.9198	0.7548	0.3027	0.147*	0.576 (2)
H27B	1.0163	0.7271	0.2992	0.147*	0.576 (2)
H27C	0.9371	0.6756	0.2672	0.147*	0.576 (2)
C26	1.0639 (6)	0.7110 (7)	0.087 (2)	0.074 (3)	0.576 (2)
H26A	1.0806	0.7151	0.0031	0.111*	0.576 (2)
H26B	1.0632	0.6618	0.1104	0.111*	0.576 (2)
H26C	1.1055	0.7360	0.1429	0.111*	0.576 (2)
S1	0.95576 (17)	0.74864 (13)	0.0961 (2)	0.0599 (5)	0.576 (2)
O5	0.8885 (11)	0.6987 (5)	0.0345 (17)	0.073 (5)	0.576 (2)
C27'	0.9400 (18)	0.7406 (10)	0.2440 (14)	0.098 (3)	0.424 (2)
H27D	0.9102	0.7137	0.3036	0.147*	0.424 (2)
H27E	0.8995	0.7731	0.2012	0.147*	0.424 (2)
H27F	0.9874	0.7667	0.2867	0.147*	0.424 (2)
C26'	1.0592 (10)	0.7461 (8)	0.080 (3)	0.074 (3)	0.424 (2)
H26D	1.0844	0.7296	0.0062	0.111*	0.424 (2)
H26E	1.1050	0.7539	0.1452	0.111*	0.424 (2)
H26F	1.0282	0.7897	0.0618	0.111*	0.424 (2)
S1'	0.9837 (2)	0.68004 (18)	0.1302 (3)	0.0599 (5)	0.424 (2)
O5'	0.9125 (17)	0.6873 (8)	0.027 (2)	0.073 (5)	0.424 (2)

### Atomic displacement parameters (Å^2^)

**Table:**

	*U*^11^	*U*^22^	*U*^33^	*U*^12^	*U*^13^	*U*^23^
C1	0.073 (5)	0.080 (5)	0.129 (7)	−0.032 (4)	0.042 (4)	−0.012 (4)
C2	0.045 (3)	0.039 (3)	0.057 (3)	0.002 (2)	0.016 (3)	0.007 (3)
C3	0.063 (4)	0.057 (3)	0.064 (4)	0.002 (3)	0.036 (3)	0.008 (3)
C4	0.078 (4)	0.063 (4)	0.049 (3)	−0.003 (3)	0.037 (3)	−0.001 (3)
C5	0.061 (4)	0.057 (3)	0.044 (3)	0.004 (3)	0.019 (3)	−0.004 (3)
C6	0.045 (3)	0.036 (3)	0.035 (3)	0.005 (2)	0.008 (2)	0.002 (2)
C7	0.040 (3)	0.041 (3)	0.039 (3)	0.010 (2)	0.010 (2)	0.007 (2)
C8	0.042 (3)	0.035 (3)	0.039 (3)	0.006 (2)	0.004 (2)	−0.003 (2)
C9	0.040 (3)	0.029 (2)	0.033 (3)	−0.005 (2)	0.002 (2)	−0.0033 (19)
C10	0.065 (3)	0.035 (3)	0.039 (3)	−0.004 (2)	0.010 (2)	−0.005 (2)
C11	0.077 (4)	0.029 (3)	0.045 (3)	−0.011 (3)	0.004 (3)	−0.008 (2)
C12	0.057 (3)	0.047 (3)	0.046 (3)	−0.019 (3)	0.008 (3)	−0.004 (2)
C13	0.052 (3)	0.041 (3)	0.040 (3)	−0.009 (2)	0.010 (2)	0.001 (2)
C14	0.038 (3)	0.033 (2)	0.031 (2)	−0.006 (2)	0.004 (2)	0.0015 (19)
C15	0.033 (3)	0.035 (2)	0.031 (2)	−0.0024 (19)	0.0039 (19)	0.0039 (19)
C16	0.040 (3)	0.030 (2)	0.030 (2)	0.005 (2)	0.003 (2)	−0.0005 (19)
C17	0.043 (3)	0.045 (3)	0.036 (3)	0.007 (2)	0.004 (2)	0.001 (2)
C18	0.049 (3)	0.050 (3)	0.048 (3)	0.015 (3)	0.004 (2)	−0.010 (3)
C19	0.057 (3)	0.033 (3)	0.038 (3)	0.011 (2)	0.001 (2)	−0.004 (2)
C20	0.048 (3)	0.030 (2)	0.034 (3)	0.001 (2)	−0.001 (2)	0.000 (2)
C25	0.079 (5)	0.074 (4)	0.052 (4)	0.005 (4)	−0.002 (3)	−0.002 (3)
C23	0.150 (7)	0.088 (6)	0.076 (5)	−0.002 (5)	0.007 (5)	−0.013 (4)
C24	0.075 (5)	0.100 (6)	0.103 (6)	0.013 (4)	0.018 (4)	0.044 (5)
C21	0.045 (3)	0.032 (3)	0.026 (2)	0.004 (2)	0.004 (2)	0.0034 (19)
C22	0.073 (4)	0.033 (3)	0.083 (4)	−0.004 (3)	0.004 (3)	−0.012 (3)
N1	0.039 (2)	0.031 (2)	0.036 (2)	0.0011 (17)	0.0061 (17)	−0.0043 (17)
N2	0.042 (2)	0.029 (2)	0.031 (2)	−0.0039 (17)	0.0049 (17)	−0.0003 (16)
N3	0.074 (3)	0.067 (3)	0.050 (3)	0.005 (3)	0.003 (3)	0.007 (3)
O3	0.045 (2)	0.0313 (17)	0.049 (2)	−0.0054 (14)	0.0186 (15)	−0.0070 (15)
O2	0.057 (2)	0.0389 (19)	0.043 (2)	−0.0104 (15)	0.0244 (16)	−0.0070 (15)
O1	0.052 (2)	0.056 (2)	0.077 (3)	−0.0158 (19)	0.0308 (19)	−0.002 (2)
O6	0.084 (3)	0.107 (4)	0.084 (3)	0.009 (3)	0.027 (3)	0.005 (3)
O4	0.066 (2)	0.0349 (19)	0.057 (2)	−0.0089 (17)	0.0159 (18)	−0.0100 (17)
Zn1	0.0403 (3)	0.0300 (3)	0.0366 (3)	−0.0041 (2)	0.0101 (2)	−0.0017 (2)
Pb1	0.04989 (13)	0.04072 (11)	0.04396 (12)	−0.01222 (9)	0.01203 (8)	−0.00356 (9)
Cl1	0.0783 (10)	0.0622 (9)	0.0613 (9)	−0.0068 (8)	0.0069 (7)	0.0132 (7)
Cl2	0.0503 (8)	0.0548 (8)	0.0523 (8)	−0.0035 (6)	−0.0015 (6)	0.0091 (6)
C27	0.103 (4)	0.106 (4)	0.083 (4)	−0.004 (3)	0.006 (3)	−0.001 (3)
C26	0.058 (5)	0.074 (11)	0.085 (6)	0.017 (7)	−0.017 (4)	0.003 (11)
S1	0.0670 (14)	0.0486 (11)	0.0625 (13)	−0.0105 (10)	−0.0049 (10)	0.0059 (10)
O5	0.057 (10)	0.099 (5)	0.063 (3)	−0.039 (6)	0.007 (6)	−0.021 (4)
C27'	0.103 (4)	0.106 (4)	0.083 (4)	−0.004 (3)	0.006 (3)	−0.001 (3)
C26'	0.058 (5)	0.074 (11)	0.085 (6)	0.017 (7)	−0.017 (4)	0.003 (11)
S1'	0.0670 (14)	0.0486 (11)	0.0625 (13)	−0.0105 (10)	−0.0049 (10)	0.0059 (10)
O5'	0.057 (10)	0.099 (5)	0.063 (3)	−0.039 (6)	0.007 (6)	−0.021 (4)

### Geometric parameters (Å, °)

**Table:**

C1—O1	1.427 (6)	C25—H25	0.9300
C1—H1A	0.9600	C23—N3	1.436 (8)
C1—H1B	0.9600	C23—H23A	0.9600
C1—H1C	0.9600	C23—H23B	0.9600
C2—O1	1.367 (6)	C23—H23C	0.9600
C2—C3	1.372 (7)	C24—N3	1.443 (7)
C2—C7	1.422 (6)	C24—H24A	0.9600
C3—C4	1.365 (8)	C24—H24B	0.9600
C3—H3	0.9300	C24—H24C	0.9600
C4—C5	1.362 (7)	C21—O3	1.319 (5)
C4—H4	0.9300	C22—O4	1.428 (5)
C5—C6	1.412 (6)	C22—H22A	0.9600
C5—H5	0.9300	C22—H22C	0.9600
C6—C7	1.406 (6)	C22—H22B	0.9600
C6—C8	1.446 (6)	Zn1—N1	2.070 (4)
C7—O2	1.312 (5)	Zn1—N2	2.077 (3)
C8—N1	1.290 (5)	Zn1—O3	2.028 (3)
C8—H8	0.9300	Zn1—O2	2.014 (3)
C9—C10	1.372 (6)	Zn1—Cl2	2.2686 (14)
C9—C14	1.406 (6)	Pb1—O1	2.791 (4)
C9—N1	1.419 (5)	Pb1—O2	2.476 (3)
C10—C11	1.379 (6)	Pb1—O3	2.420 (3)
C10—H10	0.9300	Pb1—O4	2.667 (3)
C11—C12	1.379 (7)	Pb1—O5	2.741 (5)
C11—H11	0.9300	Pb1—O5'	2.746 (7)
C12—C13	1.369 (6)	Pb1—Cl1	2.6088 (15)
C12—H12	0.9300	C27—S1	1.829 (6)
C13—C14	1.388 (6)	C27—H27A	0.9600
C13—H13	0.9300	C27—H27B	0.9600
C14—N2	1.422 (5)	C27—H27C	0.9600
C15—N2	1.292 (5)	C26—S1	1.809 (6)
C15—C16	1.441 (6)	C26—H26A	0.9600
C15—H15	0.9300	C26—H26B	0.9600
C16—C21	1.409 (6)	C26—H26C	0.9600
C16—C17	1.418 (6)	S1—O5	1.506 (5)
C17—C18	1.349 (6)	C27'—S1'	1.838 (7)
C17—H17	0.9300	C27'—H27D	0.9600
C18—C19	1.402 (7)	C27'—H27E	0.9600
C18—H18	0.9300	C27'—H27F	0.9600
C19—C20	1.359 (6)	C26'—S1'	1.809 (6)
C19—H19	0.9300	C26'—H26D	0.9600
C20—O4	1.375 (5)	C26'—H26E	0.9600
C20—C21	1.421 (6)	C26'—H26F	0.9600
C25—O6	1.230 (7)	S1'—O5'	1.492 (7)
C25—N3	1.314 (7)		
			
O1—C1—H1A	109.5	H24A—C24—H24C	109.5
O1—C1—H1B	109.5	H24B—C24—H24C	109.5
H1A—C1—H1B	109.5	O3—C21—C16	124.6 (4)
O1—C1—H1C	109.5	O3—C21—C20	118.0 (4)
H1A—C1—H1C	109.5	C16—C21—C20	117.4 (4)
H1B—C1—H1C	109.5	O4—C22—H22A	109.5
O1—C2—C3	125.5 (5)	O4—C22—H22C	109.5
O1—C2—C7	113.5 (4)	H22A—C22—H22C	109.5
C3—C2—C7	121.0 (5)	O4—C22—H22B	109.5
C4—C3—C2	120.7 (5)	H22A—C22—H22B	109.5
C4—C3—H3	119.6	H22C—C22—H22B	109.5
C2—C3—H3	119.6	C8—N1—C9	120.7 (4)
C5—C4—C3	120.6 (5)	C8—N1—Zn1	127.6 (3)
C5—C4—H4	119.7	C9—N1—Zn1	111.1 (3)
C3—C4—H4	119.7	C15—N2—C14	122.1 (4)
C4—C5—C6	120.5 (5)	C15—N2—Zn1	127.4 (3)
C4—C5—H5	119.8	C14—N2—Zn1	110.5 (3)
C6—C5—H5	119.8	C25—N3—C23	119.7 (6)
C7—C6—C5	119.8 (4)	C25—N3—C24	121.6 (6)
C7—C6—C8	123.9 (4)	C23—N3—C24	118.7 (6)
C5—C6—C8	116.3 (4)	C21—O3—Zn1	128.2 (3)
O2—C7—C6	125.0 (4)	C21—O3—Pb1	127.8 (3)
O2—C7—C2	117.8 (4)	Zn1—O3—Pb1	103.89 (12)
C6—C7—C2	117.2 (4)	C7—O2—Zn1	129.2 (3)
N1—C8—C6	125.2 (4)	C7—O2—Pb1	127.3 (3)
N1—C8—H8	117.4	Zn1—O2—Pb1	102.38 (12)
C6—C8—H8	117.4	C2—O1—C1	119.2 (4)
C10—C9—C14	119.8 (4)	C20—O4—C22	118.9 (4)
C10—C9—N1	125.1 (4)	C20—O4—Pb1	118.5 (3)
C14—C9—N1	115.1 (4)	C22—O4—Pb1	122.6 (3)
C9—C10—C11	120.0 (5)	O2—Zn1—O3	81.97 (12)
C9—C10—H10	120.0	O2—Zn1—N1	88.26 (13)
C11—C10—H10	120.0	O3—Zn1—N1	140.59 (14)
C10—C11—C12	120.3 (4)	O2—Zn1—N2	144.80 (14)
C10—C11—H11	119.8	O3—Zn1—N2	87.68 (13)
C12—C11—H11	119.8	N1—Zn1—N2	78.68 (14)
C13—C12—C11	120.4 (5)	O2—Zn1—Cl2	108.35 (10)
C13—C12—H12	119.8	O3—Zn1—Cl2	109.75 (10)
C11—C12—H12	119.8	N1—Zn1—Cl2	109.57 (11)
C12—C13—C14	120.1 (4)	N2—Zn1—Cl2	106.82 (10)
C12—C13—H13	120.0	O3—Pb1—O2	65.53 (10)
C14—C13—H13	120.0	O3—Pb1—Cl1	92.31 (8)
C13—C14—C9	119.3 (4)	O2—Pb1—Cl1	93.86 (9)
C13—C14—N2	124.8 (4)	O3—Pb1—O4	61.32 (10)
C9—C14—N2	115.8 (4)	O2—Pb1—O4	126.34 (10)
N2—C15—C16	125.1 (4)	Cl1—Pb1—O4	81.62 (8)
N2—C15—H15	117.5	O3—Pb1—O5	143.8 (5)
C16—C15—H15	117.5	O2—Pb1—O5	150.6 (5)
C21—C16—C17	119.3 (4)	Cl1—Pb1—O5	86.06 (18)
C21—C16—C15	124.0 (4)	O4—Pb1—O5	82.8 (5)
C17—C16—C15	116.6 (4)	O3—Pb1—O5'	151.5 (8)
C18—C17—C16	121.5 (5)	O2—Pb1—O5'	142.5 (8)
C18—C17—H17	119.3	Cl1—Pb1—O5'	90.82 (17)
C16—C17—H17	119.3	O4—Pb1—O5'	91.2 (8)
C17—C18—C19	119.7 (5)	O5—Pb1—O5'	9.1 (12)
C17—C18—H18	120.1	O5—S1—C26	109.0 (10)
C19—C18—H18	120.1	O5—S1—C27	102.6 (9)
C20—C19—C18	120.2 (4)	C26—S1—C27	90.6 (8)
C20—C19—H19	119.9	S1—O5—Pb1	153.7 (5)
C18—C19—H19	119.9	S1'—C27'—H27D	109.5
C19—C20—O4	124.2 (4)	S1'—C27'—H27E	109.5
C19—C20—C21	121.8 (4)	H27D—C27'—H27E	109.5
O4—C20—C21	114.0 (4)	S1'—C27'—H27F	109.5
O6—C25—N3	125.9 (6)	H27D—C27'—H27F	109.5
O6—C25—H25	117.0	H27E—C27'—H27F	109.5
N3—C25—H25	117.0	S1'—C26'—H26D	109.5
N3—C23—H23A	109.5	S1'—C26'—H26E	109.5
N3—C23—H23B	109.5	H26D—C26'—H26E	109.5
H23A—C23—H23B	109.5	S1'—C26'—H26F	109.5
N3—C23—H23C	109.5	H26D—C26'—H26F	109.5
H23A—C23—H23C	109.5	H26E—C26'—H26F	109.5
H23B—C23—H23C	109.5	O5'—S1'—C26'	99.2 (11)
N3—C24—H24A	109.5	O5'—S1'—C27'	98.7 (16)
N3—C24—H24B	109.5	C26'—S1'—C27'	92.7 (12)
H24A—C24—H24B	109.5	S1'—O5'—Pb1	112.7 (5)
N3—C24—H24C	109.5		
